# Low potency toxins reveal dense interaction networks in metabolism

**DOI:** 10.1186/s12918-016-0262-7

**Published:** 2016-02-20

**Authors:** William Bains

**Affiliations:** Earth, Atmospheric and Planetary Sciences Department, MIT, 77 Mass Avenue, Cambridge, MA 02139 USA; Rufus Scientific Ltd., 37 The Moor, Melbourn, Royston, Herts SG8 6ED UK

**Keywords:** Toxicity, Metabolic network, Protein structure, Ecotoxicology, Protein binding, Non-specific binding, Xenobiotic

## Abstract

**Background:**

The chemicals of metabolism are constructed of a small set of atoms and bonds. This may be because chemical structures outside the chemical space in which life operates are incompatible with biochemistry, or because mechanisms to make or utilize such excluded structures has not evolved. In this paper I address the extent to which biochemistry is restricted to a small fraction of the chemical space of possible chemicals, a restricted subset that I call Biochemical Space. I explore evidence that this restriction is at least in part due to selection again specific structures, and suggest a mechanism by which this occurs.

**Results:**

Chemicals that contain structures that our outside Biochemical Space (UnBiological groups) are more likely to be toxic to a wide range of organisms, even though they have no specifically toxic groups and no obvious mechanism of toxicity. This correlation of UnBiological with toxicity is stronger for low potency (millimolar) toxins. I relate this to the observation that most chemicals interact with many biological structures at low millimolar toxicity. I hypothesise that life has to select its components not only to have a specific set of functions but also to avoid interactions with all the other components of life that might degrade their function.

**Conclusions:**

The chemistry of life has to form a dense, self-consistent network of chemical structures, and cannot easily be arbitrarily extended. The toxicity of arbitrary chemicals is a reflection of the disruption to that network occasioned by trying to insert a chemical into it without also selecting all the other components to tolerate that chemical. This suggests new ways to test for the toxicity of chemicals, and that engineering organisms to make high concentrations of materials such as chemical precursors or fuels may require more substantial engineering than just of the synthetic pathways involved.

## Background

The biochemistry we observe in life on Earth is an island in the chemical space of possible biochemistry. Not all possible small organic molecules are made by life, and the chemicals making up the metabolic pathways common to life are limited to a small number of classes of chemicals – aldehydes, polyols, amines, alpha amino acids etc.. Understanding why biochemistry uses the molecules that it does is central both to engineering biochemistry to produce useful products and to understanding how terrestrial biochemistry originated. Is the restriction on the observed chemistry of life simply because life has not evolved the catalysts needed to make other molecules, because life has not found a need for them, or because there is selection against chemistry outside ‘biochemical space’?

It is plausible to suggest that life simply has not invented the means to make some classes of chemicals. We know that life makes carbon-carbon bonds using aldol condensation and not (for example) metathesis [1, 2], although metathesis enzymes can be designed in principle [3]. There may simply not be any functional reason for making some molecules driving the evolution of the relevant enzymatic mechanisms.

There may also be limits on what biochemistry can achieve outside those imposed by catalytic mechanisms and the function of metabolites. For example, it has previously been shown that a simple measure of the degree of saturation of a molecule may be used to indicate that molecule’s toxicity, in the absence of *any* other structural information about the molecule, a finding that is related to the distribution of biochemicals in chemical space [4]. It would be surprising if this were the only such constraint on the molecules of life.

In this paper I present evidence that there is selection against the incorporation of chemicals that contain structural features not found in central metabolism – chemicals that I term ‘Unbiological’ – into metabolism, separate from the constraints provided by selection for specific function and the ability of life to catalyse specific types of reaction. Specifically, the sections below argue that:

i) the chemical space of the biochemicals that are common to life on Earth is a small subset of the chemical space possible to the chemistry of life ('Biochemistry occupies a limited chemical space').
ii) that chemicals outside biochemical space have a higher chance of being toxic at millimolar concentrations than chemicals that fall inside biochemical space ('Mild toxicity is correlated with ‘UnBiological’ chemical characteristics' thru 'Threshold for correlations is millimolar concentration').
iii) that a wide range of experimental data suggests that many small molecules bind to many proteins with low millimolar affinity, which provides a mechanism for the toxicity of chemicals at millimolar concentrations ('Mechanism of Ub correlation with toxicity')
iv) that the reason for correlation of the toxicity of chemicals and their distance from biochemistry is that life has systematically evolved proteins to avoid unwanted millimolar interactions with metabolites in order to avoid poisoning itself ('Proposed mechanism of correlation of Ub with millimolar toxicity').

The results in the paper are in two parts to reflect this reasoning. The Results and discussion sections (Figs. 2, 4 and 5) describes the chemical space of life and the low level toxicity of chemicals falling outside this space. The sections on Mechanism of Ub correlation with toxicity and Proposed mechanism of correlation of Ub with millimolar toxicity (Figs. 6 and 7) provides an explanation for this effect.

These results suggest that biochemistry is more of an integrated whole than the conventional metabolic map would suggest. This has theoretical and practical implications, which I discuss briefly at the end of the paper.

## Results and discussion

### Biochemistry occupies a limited chemical space

I first establish that biochemical space is a relatively small subset of the possible chemical space from which metabolism could be selected. It is a commonplace that many of the components of primary metabolism “look similar to each other” (as undergraduates learning how to distinguish the α-amino acids or the sugars of the Calvin Cycle can attest). This section establishes that this apparent limitation of metabolism to a few chemical types is a real restriction in chemical space.

**Fig. 1:**
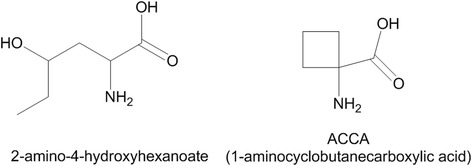
Examples of non-metabolites

The chemical space from which metabolism is selected is the space of chemicals made from C, N, O, and H, with S as S(II) and P(V), bonded in ways that are found in biological molecules. For example, 2-amino-4-hydroxyhexanoate looks like a plausible amino acids, but it happens not be made by life,^1^ whereas ACCA (Fig. 1) does not fit an intuitive feeling of what a biochemical looks like, as few biological compounds contain a cyclobutane ring. Of the myriad compounds that can be formed from the elements C, N, O, P and S (and H), life rarely forms hydrazines, peroxides, rings of less than five atoms, or phosphorus compounds other than phosphates.

These rules, and some others relating to molecular stability, were implemented in the program Combimol as previously described [5]. The chemical space of possible biochemicals includes structures usually excluded from drug design due to their sensitivity to metabolism [6]. From the chemical space of all such molecules, all Fragments were generated as described in [7, 8]. This provides a library of Substructures with which to probe the space of actual molecules that make up metabolism.

Life’s metabolic diversity is enormous [9]. For the purposes of this paper, I use a small subset of metabolites that are components of the central metabolic processes of all life on Earth, and pragmatically those processes that are shown on the Roche/Expasy metabolic map [10]. All the small molecules listed in Part 1 (“Metabolic Pathways”) were used as a set of metabolites here called “core metabolism”, a collection of 611 molecules widely used by all life on Earth (some steroid hormones were not used, as they are chemically very similar and so contribute no new chemical structural types to the data set).

**Fig. 2:**
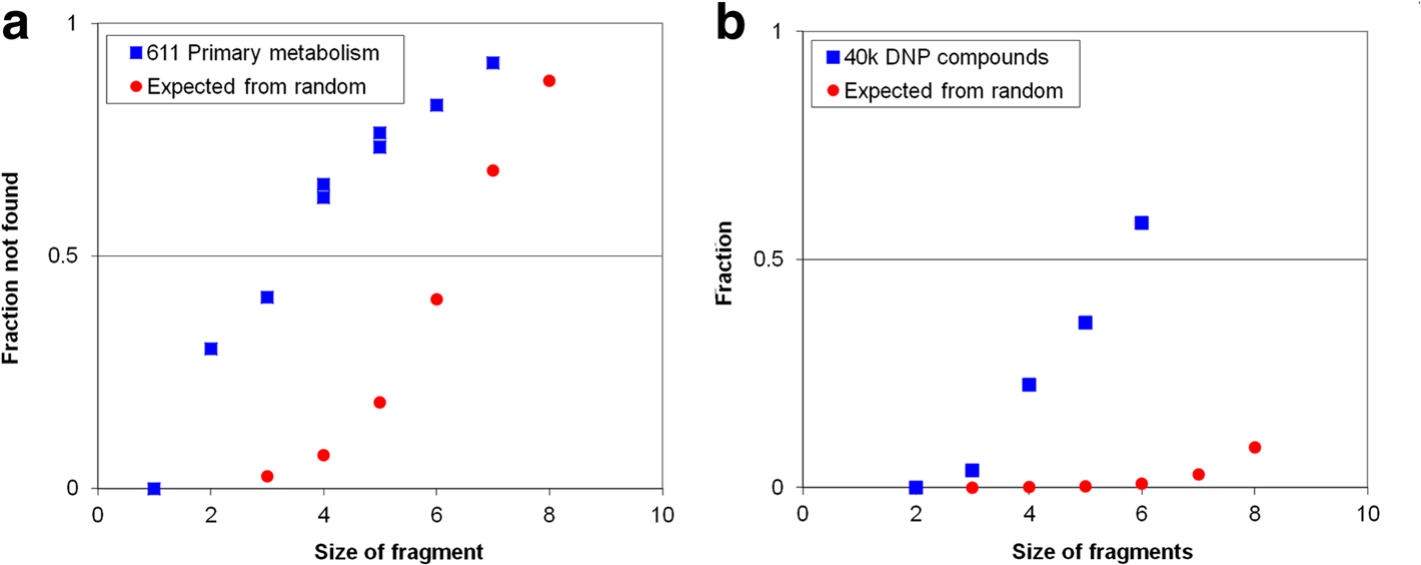
Extent of biochemical space. Fraction (*Y axis*) of Fragments derived from the space of all possible chemicals that are *not* found in actual metabolites, compared to the fraction that would be expected not to be found in the same number of chemicals sampled at random from the chemical space of possible metabolites, plotted as a function of fragment size (*N – X axis*). Blue squares – fraction of fragments not found in actual metabolites. Red circles – fraction not found in an equivalent size collection of random molecules. Panel **a**: fragments not found in the ‘core metabolism’ of 611 molecules represented in the ExPasy metabolic map. Panel **b**: Fragments not found in the ~45,000 unique molecules listed in the Dictionary of Natural Products [11]

There are more Fragments of 5, 6 and 7 atoms than there are metabolites in core metabolism, so we would expect that some of them would not be represented in that metabolism. The chances that a 5-atom Fragment will be a substructure of a molecule depends on the size of the molecule. Figure 2a shows the expected fraction of those Fragments that would *not* be found in a set of 611 molecules if the molecules were constructed randomly from the atoms and bonds found in core metabolism. (The algorithm used to estimate the frequency with which a Fragment will match a molecule selected at random from the space of chemicals is described in more detail in Appendix 1.) Fig. 2a shows that the expected number of Fragments that are not found is substantially smaller than the actual number: core metabolism must represent a small subset of the chemical space of possible water-stable chemicals that can be made from C, N, O S(II) and P (V). Figure 2b extends this analysis to the ~45,000 natural product chemicals in the Dictionary of Natural Products (DNP - [11]). DNP records the detection and structural analysis of organic chemicals from any natural source, and so samples the full diversity of chemistry of terrestrial life. If terrestrial biochemistry sampled all the chemical space of CHON, S(II) and P(V) chemistry, then essentially all of the Fragments searched here should be represented in the database. However over 50 % of 6-atom Fragments are not found in the database.

Why is biochemistry apparently limited to a subset of the possible chemistry that life could perform? The next three sections demonstrates that chemicals that fall outside the chemical space occupied by biochemistry are not merely unlikely to be part of a metabolism, but interfere with that metabolism in a way as to produce a toxic effect, and the further outside ‘biochemical space’ they are, the greater that antagonism.

### Mild toxicity is correlated with ‘UnBiological’ chemical characteristics

In this section I introduce a measure of how different a chemical is from the chemical space of life. I show that a greater difference is correlated with low levels of non-specific toxicity. Toxicity is related to the existence of structures in the test chemical that are different from chemical structures usually found in biology.

Chemicals can be toxic for one of three broad reasons.

Toxic chemicals can be chemically reactive, such as formaldehyde or mercury compounds, and so chemically modify the components of life. Reactive toxicity depends on specific chemical functionality. The Combimol chemical generation software automatically excludes reactive moieties, and so this is not a class of toxicity probed by these studies.

Toxic chemicals can interact with a specific molecular mechanism in the organism, and so disrupt a particular biochemical function (see discussion in [12–17]). Drugs and plant secondary metabolite toxins achieve their effect in this way. This is caused by very specific chemical structures, which confer specific toxicity on molecules that contain them. Such ‘structural alerts’ were originally identified to predict mutagenicity [18], but have been extended to more general toxicity prediction in programs such as DEREK [19], TOPKAT, MULTICASE [20] and others [21]. In my terms, a ‘structural alert’ is a Fragment that has a high affinity for a specific molecular target whose blockade produces a toxic effect. In agreement with this, ‘structural alert’ approaches to toxicity prediction or other structure-activity relationship methods that try to relate large structural features to biological endpoints work well for specific toxicity mechanisms, such as HERG blockade giving rise to cardiac toxicity [7] or electrophilic attack on DNA giving rise to carcinogenicity [18].

Structural alert approaches do not work well for predicting broad toxicity endpoints, such as death [22]. A wide range of industrial chemicals have, or are claimed to have, toxicity that is not severe or life-threatening at low concentrations, and which is not obviously linked to structural alerts, but which nevertheless cause morbidity and mortality in model organisms at higher concentrations. Interactions of some of these chemicals with various receptors or enzymes is claimed, but most are simply observed to disable or kill model species without a mechanism for their toxicity being known or postulated. It is this third class of low potency, non-specific toxicity that I have probed further below.

I use a Fragment-based approach to identify the largest part of a molecule which is different from anything found in biology. Fragment-based methods of describing molecules are well known, computationally simple approaches to describing a molecule in terms of how its structure would be drawn by a chemist [23]. Several groups have described using a fragment-based approach for molecular description and design [24–26], claiming that building drug-like molecules from chemical fragments derived from biochemicals lead to more ‘drug-like’ results.

**Fig. 3:**
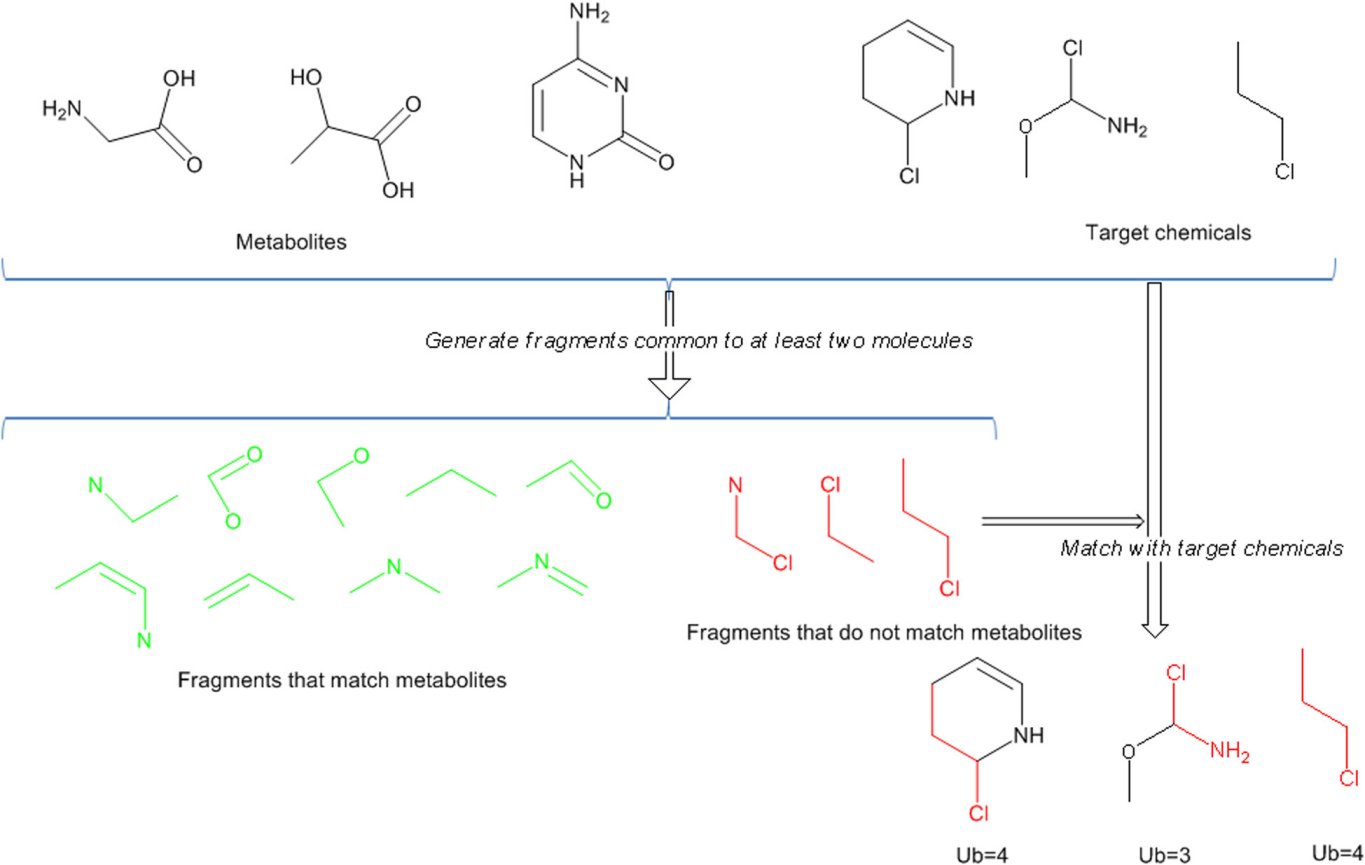
Cartoon of calculating UnBiological (*Ub*). This takes a ‘toy domain’ of four metabolites and three target molecules to explain the process. Only fragments of 3 or 4 atoms are considered in this example. In reality there are 611 metabolites, ~5000 targets molecules (note that a number of molecules are tested in more than one experimental series in Table 3) and 30912 Fragments of size 3 to 14 atoms. Metabolites and target molecules are used to generate Fragments that are present in at least two of the overall set of molecules (This is a convenient limitation on the number of Fragments, and may be revised in future implementations of the algorithm). Fragments are classified as to whether they occur in the set of metabolites (*green*) or do not occur in metabolites (*red*). The target set of molecules is then matched to the set of Fragments that do not occur in metabolites – the size of the largest such Fragment is the Ub measure. Note that in this simplified model it is clear that the presence of a chlorine atom confers ‘*UnBiological-ness’* on a molecule. The size of the *Ub* fragment can be the same as the size of the whole molecule (e.g. 1-cloropropane in this example). As illustrated here, this approach takes no account of the potential reactivity of a molecule, only its topological structure

I define a measure of the fraction of a molecule that is not similar to a biological molecule, which I call “*UnBiological*” (*Ub*). *Ub* is defined as the largest region of a test molecule that does not overlap with at least one molecule in the core metabolite set. *Ub* has to be defined in terms of the size of the Fragments used to determine overlaps. Because the difference between the expected and observed occupancy of chemical space shown in Fig. 2 is greatest for 5-atom and 6-atom Fragments, 5-atom and 6-atom overlaps were both used for this study, designated as *Ub*_*5*_ and *Ub*_*6*_ respectively. The algorithm used to generate the *Ub* measures is summarised in the Methods section, with more detail on the actual computational steps used in Appendix 2, and a graphic summary of the process in Fig. 3.

In Table 1 I show the result of correlating the values of *Ub* calculated for each chemical species with the toxicity endpoints measured for that chemical species, for a variety of measures of general, non-specific toxicity. As is standard in toxicology, the measure of toxicity is the logarithm of the concentration that gives a half-maximal toxic effect in the system under consideration. Log (concentration) scales are commonly used in biochemistry because of the linear relationship between the binding energy of a small molecule binding to a large one and the logarithm of the equilibrium constant of that binding. Pragmatically, a log scale also enables visualization of data spanning many orders of magnitude. The half-maximal affect (EC_50_ or LD_50_) is the commonly reported value for many toxicological and pharmacological measures. In the case where the effect is causes by simple binding to a single target, a half-maximal effect represents the concentration at which the target is 50 % occupied, i.e. the Kd.

**Table 1:** Correlations of *Ub* with toxicity endpoints

Endpoint	Number	Ub_5_	Ub_6_
Trout 24 h	186	−0.230**	−0.337***
Trout 96 h	181	−0.419***	−0.516***
Pteronarcys (24 h)	52	−0.433**	−0.385**
Pteronarcys (96 h)	52	−0.456***	−0.369**
Bluegill (24 h)	157	−0.149	−0.215**
Bluegill (96 h)	172	−0.216**	−0.276***
Gammarus (24 h)	113	−0.437***	−0.208*
Gammarus (96 h)	132	−0.407***	−0.205*
Fathead minnow	578	−0.311***	−0.308***
Rat oral	814	−0.441***	−0.372***
Mouse oral	398	−0.199***	−0.191***
Rat IP	170	−0.214**	−0.147
Mouse IP	290	−0.180**	−0.161**
AMES (mutagenicity)	163	−0.316***	−0.518***
CPDBAS rat	519	−0.198***	−0.191***
CPDBAS mouse	402	−0.145**	−0.198***
CPDBAS hamster	44	−0.430**	−0.351*
Drosophila	139	−0.397***	−0.337***
Lemna - non-Herbicides	149	−0.428***	−0.502***
Lemna - Herbicides	174	−0.392***	−0.428***
Tetrahymena	334	−0.408***	−0.448***
Chlorella	91	−0.578***	−0.738***
Scenedesmus	63	−0.237	−0.467***
Yeast	253	0.095	−0.014
NCI	768	−0.113**	−0.137***

Rank Correlation coefficient between toxicity endpoints and *UnBiological (Ub)* measures. Two *Ub* measures are shown – *Ub*_*5*_ and *Ub*_*6*_, calculated from an overlap of 5 and 6 atoms between target molecule and the pool of metabolites. See Appendix 2 for more detailed descriptions of calculation of *Ub*. Column 1: toxicity endpoint. Column 2: number of data points. Column 3 and 4: correlation of *Ub*_*5*_ and *Ub*_*6*_ respectively with appropriate toxicity endpoint. Significance of the correlation of flagged by asterisks.* = *p* < 0.05.** = *p* < 0.01*** = *p* < 0.000714. Note that*** is a value selected to be 0.05/(35*2), to correct for multiple testing of 35 toxicity endpoints and 2 correlates. If *Ub*_*5*_ and *Ub*_*6*_ were randomly distributed with respect to toxicity, then we would expect to have to do this study 20 times to come up with *one* correlation of *p* < 0.000714

Here the *Ub* measure (i.e. how much of a molecule *does not* match a structure found in core metabolism) is negatively correlated with the logarithm of the concentration at which a chemical has a half-maximal effect. This might be LD_50_ for a lethal toxicity measure or EC_50_ for a non-lethal measure. A negative value of the correlation means that a larger *Ub* is associated with a lower concentration, i.e. with a more potent toxin.

In almost all cases, for *Ub*_*5*_ and *Ub*_*6*_, there is a significant negative correlation between *Ub* and toxic concentration. For a wide range of living systems, from isolated mammalian cells through unicellular plants and protests to multicellular plants and diverse animal species, *Ub* is correlated with toxicity. This correlation is highly statistically significant. The “***” level of significance in Table 1 is an indication of *p* < 0.000714 that the indicated correlation will be produced by chance. There were around 70 correlations performed for this initial analysis of the data (35 data sets, including two not shown sub-dividing the rat and mouse data into pharmacological categories, which had little effect, correlated with 2 *Ub* endpoints). If *Ub* was uncorrelated with toxicity, there is only a *p* = 0.05 chance that we would observe *one* “***” level correlation in this data set.

The one exception to the pattern of correlation of *Ub* with toxicity is *Saccharomyces cereviseae*, which shows only weak correlation between the toxicity endpoints reported here and Ub_5_ or Ub_6_. A possible reason for this will be discussed below in the section on thresholds for correlations.

I emphasize what this does and does not show. The correlations show robustly that molecules with segments that are not represented in the chemicals of core metabolism have a higher chance of being toxic at any given concentration level than molecules made up of structures found in core metabolism. The larger that “*UnBiological*” segment is, the more toxic the molecule is. None of the molecules tested for toxicity here are normal components of central metabolism (arguably with the exception of ethanol).

However this is not a method for detecting or predicting pharmacology mediated by a single, known target, or for detecting or predicting toxicity based on a single mechanism. The effects being detected here are relatively non-specific: while many of the toxins are known to interact with proteins, they typically interact with many proteins, and toxic effects often cannot be attributed to a specific molecular interaction. This is illustrated by the exploratory analysis in Table 2. Table 2 shows the result of correlating *UnBiological* with three conventional toxicity endpoints and two pharmacological ones. HERG toxicity is a significant risk factor for cardiac toxicity in pre-clinical drug candidates, and is detected by screening for blockade of the HERG ion channel in cells [7, 27]. Oestrogenic potential is a common ecotoxicological toxicity measure, and is measured here by binding to the oestrogen receptor [28]. Tadpole narcosis is a whole organism measure of both Central Nervous System penetration and effect on a select set of neurotransmitter receptors [29]. All three are therefore mechanism-based measures of toxicity, and all three show weaker correlations with *Ub*_*6*_ and no correlation with *Ub*_*5*_. An initial statistical analysis of the distribution of Ub_5_ and Ub_6_ in the molecules used for the analyses in Tables 3 and 4 (see Appendix 3) suggests that the molecules analysed for Tadpole Narcosis may be atypical of the other sets in the study, and so the lack of correlation found between Ub_5_ and Tadpole narcosis may be a result of an unrepresentative set of chemicals. The other sets of chemicals whose analysis is summarized in Table 4 appear similar in overall Ub_5_ and Ub_6_ properties to those whose analysis is summarized in Table 3.

**Table 2:** Correlation of *Ub* with other biological endpoints

Endpoint	Number	UB_5_	Ub_6_
HERG	229	−0.062	0.179**
Oestrogenic	131	−0.024	−0.342***
Tadpole narcosis	141	−0.043	−0.267**
COX-2	107	−0.069	−0.149
Antihistamine	61	−0.097	−0.0126

Rank correlation coefficient of three target-related toxicity measures and two pharmacological endpoints with UnBiological measures *Ub*_*5*_ and *Ub*_*6*_. Column 1: Pharmacological endpoint. Column 2: number of data points. Columns 3 and 4: correlations with *Ub*_*5*_ and *Ub*_*6*_ respectively. Significance flags are the same as in Table 1

**Table 3:** Biological datasets

Data set	Number of compounds	Species	Measured endpoint	Source	
Whole organism toxicity endpoints					
Trout (24 h)	186	Oncorhynchus mykiss	Death	[111]	These two data sets differ only in the time of exposure – 1 and 3 days
Trout (96 h)	181				
Pteronarcys (24 h)	52	Pteronarcys californica	Death	[111]	These two data sets differ only in the time of exposure – 1 and 3 days
Pteronarcys (96 h)	52				
Bluegill (24 h)	157	Lepomis macrochirus	Death	[111]	These two data sets differ only in the time of exposure – 1 and 3 days
Bluegill (96 h)	172				
Gammarus (24 h)	113	Combined data from G. fasciatus, G. lacustris and G. Pseudolimnaeus	Death	[111]	These two data sets differ only in the time of exposure – 1 and 3 days
Gammarus (96 h)	132				
Fathead minnow	578	Pimephales promelas	Death	[112]	
Rat oral	814	Rattus norvegicus	Death	[113]	Rodent toxicity data was manually curated from The Merck Index. Note that ‘molar’ values for mammalian whole organism studies are calculated as moles/kg body mass
Mouse oral	398	Mus musculus			
Rat IP	170	Rattus norvegicus			
Mouse IP	290	Mus musculus			
AMES (mutagenicity)	163	Salmonella typhimurium	Mutated colony formation	Data collected and provided by Choracle Ltd, derived from Toxnet [114]	
CPDBAS rat	519	Rattus norvegicus	Tumour formation frequency	[115]	
CPDBAS mouse	402	Mus musculus			
CPDBAS hamster	44	Mesocricetus auratus			
Drosophila	139	Drosophila melanogaster	Death	[116]	Only compounds with at least two compound concentrations reported included
Lemna - non-Herbicides	149	Lemna gibba and Lemna minor	lack of growth/leaflet reduction	[117–136]	Compounds developed for reasons other than their herbicide effect
Lemna - Herbicides	174	Lemna gibba and Lemna minor	lack of growth/leaflet reduction	[117]	Compounds developed as herbicides (primarily for macroscopic land plants)
Tetrahymena	334	Tetrahymena pyriformis	Death	[137–140]	
Chlorella	91	Chlorella vulgaris	Death	[141]	
Scenedesmus	63		Cell numbers (combination growth inhibition and death)	[142–154]	Data-set heavy on chlorinated and nitrated aromatic compounds
Yeast	253	Saccharomyces cereviseae	Growth inhibition	[106]	Mostly drug-like molecules: See methods section for details of this analysis
Other endpoints					
NCI	768	Homo sapiens	Cell number (cell growth vs. cell killing)	[39]	Cell culture assay, not whole organism. Cytotoxicty data from the NCI anti-HIV compounds screening programme.
HERG	229	Homo sapiens	Ion channel blockade	[7]	Ion channel assay in cloned receptor assay, not whole organism test
Oestrogenic	131	Rattus norvegicus	Receptor binding IC_50_	[155]	Receptor binding assay, not a cell- or organism-based assay
Tadpole narcosis	141	Rana temporaria	Narcosis (reversible lack of motion)	[29]	
COX-2	107	N/A	Cycloxygenase-2 inhibition	[156]	
Antihistamine	61	N/A	Histamine receptor blockade	[157–159]	A variety of related structures, including anti-psychotics

Data sets used in this paper

**Table 4:** Structures sets used for docking

Protein	PDB structural data sets used for docking
ABL	2e2b 1 m52 1iep 3k5v 3qri 3qrk 3g6g 1ab2 2g2h 2hiw 2gqg 2hz0 3cs9
Aldolase	1ald 2ald 4ald
HIV protease	1a94 1kj4 2bpz 2qhz 2qi6 2r5p 2r5q
PDE2b4b	1f0j 1ro6 1ro9 1ror 2qyl 3frg 3gwt 3hmv 3o57
PPAR	1i7g 1kkq 2npa 2p54 2rew 2znn 3et1 3kdu

Drugs can also be toxic in the wrong place or dose. In Table 2 I also show that *Ub* is uncorrelated with the potency of compounds for just two drug targets (for which I had data to hand), antihistamines and NSAIDs that inhibit cycloxygenase-2. Neither show any significant correlation with *Ub*.

I do not claim that other toxicity or pharmacology endpoints will not be found to correlate with *UnBiological.* The examples in Table 2 are included to make the point that *Ub* is correlated with broad, whole-organism toxicity, not necessarily with target-specific mechanisms.

### Reasons for variability of correlation

Table 1 provides robust statistical evidence for believing that *UnBiological* is correlated with whole organism toxicity. However the degree of correlation varies substantially between species, as does the statistical significance of that correlation. This could be due to genuine biological differences, or differences in the chemical space being sampled. The issue of chemical space coverage is significant. For example, an initial study suggested a strong correlation of *Ub* with the potency of phosphodiesterase-4b inhibitors (data not shown). However this was based on analysis of the data in two QSAR studies on PDE4b inhibition. The chemicals in the two studies were very similar to each other (i.e. were two specific series of chemicals). In effect, *Ub* was being used to classify compounds into the two studies, one of which was developing a much more potent drug series than the other. Therefore *Ub* could identify more potent PDE4b inhibitors, but for the trivial reason that it was identifying two studies looking at two classes of chemicals. When a wider set of PDE4 inhibitors was analysed, the correlation was reduced.^2^ It seems likely that, as with other QSAR methods, *UnBiological* will work best on a chemical set spread uniformly across the chemical space that is to be analysed. Bias in the molecules that happen to have been investigated to generate the data analysed here may be a cause of the differences in correlation of *Ub* and toxicity. This can only be addressed by collecting a more systematic set of multi-species toxicity data on defined chemicals. Data filed for the REACH legislation [30] may provide such a data set in the future.

Statistical significance is a function of sample size. It is not practical to collect hundreds of toxicity endpoints from all the species involved, and not desirable to discard endpoints from species that have been extensively tested. Therefore this aspect of variability has been retained in the study.

### Threshold for correlations is millimolar concentration

**Fig. 4:**
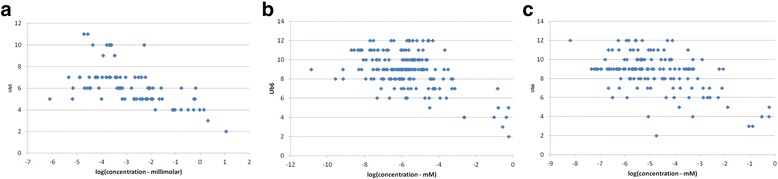
UnBiological vs. toxicity for selected organisms. Plots of *UnBiological* vs. toxicity endpoints for three of the data sets analysed here. Each dot represents the LD_50_ (*X axis*) vs *Ub* value (*Y axis*) for one compound. *Ub* is calculated as described in the Methods section and Appendix 2. In summary, Ub represents the largest region on a molecule that is not present in a metabolite, as defined by a 5-atom (*Ub*_*5*_) or 6-atom (*Ub*_*6*_) overlap. **a**: *Ub*_*5*_ vs. LD_50_ for Chlorella, **b**: *Ub6* vs. LD_50_ for Rainbow trout, **c**: *Ub*_*6*_ vs. LD_50_ for Lemna, intoxicated with compounds other than herbicides

Many of the correlations summarised in Table 1 are statistically robust but relatively small. Direct plots of *Ub* vs. toxicity are usually uninformative. A good and strong statistical correlation can be shown for data that does not appear ‘correlated’ to the eye – this is the reason for performing correlation calculations. However, for some of the more strongly correlated data sets containing relatively few data points, such as those plotted in Fig. 4, the correlation between *Ub* and toxicity appears stronger for weakly potent toxins than for highly potent ones (i.e. the correlation is clearer on the right-hand side of the graphs, and weak or non-existent on the left-hand side). This has also been observed for the correlation of structural redox with toxicity [4].

**Fig. 5:**
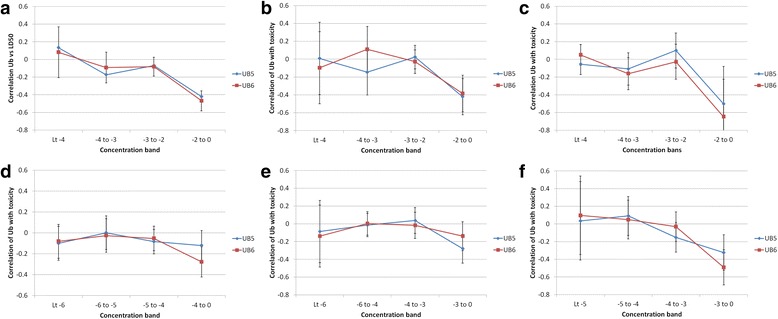
Correlation of *UnBiological* with toxicity by potency band. Correlation of *Ub*_*5*_ and *Ub*_*6*_ with different toxicity endpoints. For each data set, the data on a compound was binned for compounds having different EC_50_ or LD_50_ values, and the correlation of the toxicity endpoint with *Ub* was correlated with the toxicity values within that concentration range. Thus for Rat oral LD50 (Panel **a**), toxicity was binned into Log(LD50) < −4, Log(LD50) between −4 and −3, Log(LD50) between −3 and −2, and Log(Ld50) > −2, all values in molar. Correlations were calculated for each of these four data sets. Error bars are 95 % confidence limits for the correlation, based on the number of data points in each bin. For all panels: X axis = concentration bins, in log (*molar*). Y axis: correlation of *Ub* and toxicity within that data sub-set. Panels **a**: to **f**: − Rat oral toxicity, mouse oral toxicity, rat carcinogenic potential (from CPDBAS), NCI cell line cytotoxicity, Fathead minnow toxicity and tetrahymena toxicity

For some of the data sets there are a sufficiently large number of data points to split the data into potency bands and correlate these independently with *UnBiological*. The results from this analysis are shown in Fig. 5. For consistency, toxicity data was binned into bands of round number log units of LD_50_ or EC_50_, which results in different numbers of data points in each bin, and hence different levels of significance for the resulting correlations.

Figure 5 shows trends in most of the larger data sets that lower potency toxins have better correlation with *Ub* than higher potency toxins. For the NCI cytotoxicity and Fathead Minnow data (Fig. 5d and e respectively) there is little trend for UB_6_, although for UB_5_ only the highest concentration data (−3 to 0 band) shows a statistically significant correlation (i.e. the certainty range is below 0). For all other data sets, both UB_5_ and UB_6_ show negative correlation of *Ub* with concentration (i.e. confidence limits are <0) only for the highest concentration band. Figure 5 confirms, for these data sets, that the correlation of *UnBiological* with toxicity is an effect seen primarily in compounds that have *low* intrinsic toxicity.

This observation may explain the failure to observe a correlation of *Ub* with toxicity in *Saccharomyces*. In the data set analysed here, *Saccharomyces* was tested for the effects of chemicals at six concentrations from 1.3 uM to 100 uM. Thus no chemical with an IC_50_ of >100 uM could be detected in this screen, and so the IC_50_ values analysed here are all below the threshold at which a statistically robust correlation of *Ub* and toxicity would be expected.

## Mechanism of *Ub* correlation with toxicity

The observation that a simple and non-specific measure of chemical structure like *Ub* might be correlated with toxicity is unexpected. The observation that the correlation is more pronounced for weak toxins is, on the face of it, baffling. QSAR measures of biological potency are usually more effective for the most potent agents – whether toxins, drugs, hormones or other effectors. The findings in Figs. 4 and 5 therefore require a mechanistic explanation for the correlation of *Ub* with toxicity that operates at millimolar but not micromolar concentrations. This second part of the paper, and the results shown in it, address the plausibility of a potential mechanism.

The mechanism I propose here is that many, probably most chemicals will interact with some, maybe many, components of the cell at millimolar affinity.

The distinction of small molecules into ‘ligands’ and ‘non-ligands’ is a convenient classification for small molecules with respect to their effects on a specific protein, but it is a fiction not reflected in chemical reality. It suggests an absolute distinction between ‘binding sites’ and ‘non-binding’ sites. In reality, a small molecule can interact with atoms across the surface of a protein, and can often bind to proteins in more than one conformation and at more than one site (discussed further below). Only those sites which are unique and bind molecules with much higher affinity than any other site are called ‘binding sites’. The reality of the other sites that can, and do, interact weakly with small molecules is however illustrated by experimental evolution studies, where new protein functions are typically created by selecting new modes of interaction between protein and ligand from weak interactions already present in the original protein [31].

There is a substantial body of literature that suggests that many, maybe most small molecules can interact with many, possibly most proteins at millimolar concentration. I summarise three lines of such evidence below. This data will be very familiar to those involved in pharmaceutical screening programmes.

### High-throughput screen data

High-throughput screening (HTS) is a common route to discovering novel biological function in large libraries of chemicals. In an HTS campaign, a very large library of chemicals (tens to hundreds of thousands) is tested at one or a few concentrations in an entirely automated assay designed to give a simple, semi-quantitative measure of whether a chemical interacts with a specific molecular target. Compounds that reach a threshold of activity (“hits”) are then taken on for further study. Such large screening programmes are a common approach to drug discovery [32].

Typical reports of such screens report a ‘hit rate’ of between 0.1 and 1 %, and report finding ‘hits’ that bind to the target protein with micromolar affinities [32, 33]. Most freely available databases of the biological effects of molecules also assume that compounds either bind to a target with micromolar affinity or better, or that they do not [34]. Such databases imply that ‘not binding’ at the tested concentration means not binding at all. However this literature is misleading. More detailed reports of HTS campaigns routinely report widespread “non-specific” interaction of small molecules with protein or cellular targets (see e.g. [35–38]). Assay conditions, screening concentrations, detection thresholds and other factors are tuned to achieve a hit rate of <0.1 % in what in reality is a continuum of binding.

**Fig. 6:**
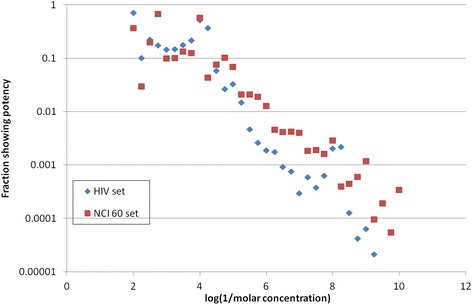
NCI screening data analysis. X axis: concentration. Y axis: fraction of compounds in NCI public datasets on cell-based screens that show inhibitory effect in that assay as a fraction of number of compounds tested at that concentration. Results are binned into concentration bins on a log scale, each bin representing log (concentration) = 0.25. Blue diamonds: HIV screening data [39]. Red squares: cell line screening for anti-cancer effect [40]

Usually the raw data behind an HTS screening programme is not available – only summary statistics and the data on the ‘hits’ is published. However the reality of HTS binding can be illustrated with HTS data available from the National Cancer Institute, which has published detailed screening data on a library of ~47,000 compounds for anti-HIV effect and ~60,000 compounds for anti-cancer effect [39, 40]. Figure 6 summarises this data in terms of the chance that a compound will be found to have a positive effect on a screen at a particular concentration. Not all compounds are tested at higher concentrations, so Fig. 6 plots the fraction of compounds that have an effect at a concentration as a fraction of the compounds tested at that concentration. The result is clear. There is a continuum of affinity in this essentially random set of chemicals for their molecular targets, and while the chance that a compound has an effect at micromolar concentration is low, the chance that it has a biological effect on mammalian cells approaches 1 as the concentration approaches 10 mM.

### Fragment-based screening

Fragment-based screening (FBS) seeks to identify small molecules that bind with relatively low affinity to proteins, and then combine these into larger molecules that bind with greater affinity (reviewed in [41–45]). FBS actively looks for high micromolar or low millimolar affinity of small molecules to proteins. It is a commonplace for researchers in this field that many small molecules (“fragments”) bind to most proteins at low millimolar concentrations. For example, [43] comment that “Novice users [of fragment-based screening by BiaCORE] are often surprised to see how often small molecules bind indiscriminately to proteins when compounds are assayed at high concentrations”. The data they give suggests ~75 % of a 1000 compound subset of the Maybridge Ro3 library bound equally well to two different targets at high micromolar to low millimolar concentrations. Congreve et al. [46] find that 90 % of compounds in their library have some binding at mM affinities. Hubbard [41] found a ‘hit’ rate of between 1.5 and 4.7 % when measuring small molecule binding to proteins at 0.5 mM by NMR. Giannetti [47] reviewed 20 different fragment-based screens, and report that all show ‘non-specific’ binding at affinities of 1 – 4 mM, although the highest affinities found ranged over three orders of magnitude in the different experiments. Spurlino [48] found that between 5 and 50 % of a library bound to target protein crystals at 5 mM (depending on library/target combination).

### Other Non-Specific Binding observations

Non-specific interactions are a fact of life for pharmaceutical researchers, even among molecules that are selected for their specificity of action. Even in launched pharmaceuticals, supposedly selected for their singular, specific interaction with one target or target class, multi-target interactions are being recognised as the rule rather than the exception [49, 50]. LaBella commented “The non-specificity of drugs is a generally acknowledged truism” over 20 years ago [50], with genome-scale testing of molecules confirming that nearly all small molecules bind to multiple proteins [51]. Such ‘non-specific effects’ now being accepted as a critical part of drugs’ actions [52, 53]. Houk et al. review a range of studies of binding of small molecules to proteins and cyclodextrin mimics of protein binding sites, and find an average binding affinity of ~0.5 mM [54].

### Molecular mechanism of low affinity binding effects

It is worthwhile touching briefly on potential mechanisms of millimolar binding of compounds to proteins, and the likelihood that this will materially affect the protein’s function. Again, we must challenge the conventional model of a ligand binding to a ‘binding site’ on a protein. Structural studies have shown that many proteins can bind a diversity of chemical structures through adaptation of their structure (reviewed in [55–57]). Many proteins exist in dynamic equilibrium with partially or completely unfolded structures, some being dominantly disordered [58–61]. Post translational modification [62] or ligand binding [63, 64] can switch proteins from a disordered to a more ordered state, switches which can be related to their regulation and function [59, 65]. Proteins can also have multiple ordered, metastable structures (reviewed in [66]), and different folding states can be selected by ligand binding and have significantly different biological function (see for example [67–71]). There can also multiple folding paths leading to each of those states (see e.g. [72]).

A molecule that binds even weakly to one folding state of a protein and not to another will bias the population of protein folding states by stabilising the bound state over the others (by definition, if a small molecule M binds to folding state A, then the combination of A + M must be more stable than A and M on their own, and hence more likely to occur). If one of the structures in the spectrum of structures has a function absent from other structures, then binding of the small molecule will change that function by changing the amount of the functional conformer. The binding need not be ‘tight’, and may not even be detectable on the canonical crystal structure for the protein, but will nevertheless affect function in the cell.

In conclusion, it is found in many types of experimental systems that all, or nearly all, small molecules interact with many proteins with low millimolar affinity, and these low affinity bindings can have significant biological affect through modulation of the population of structures adopted by a protein. This observation leads both to an explanation of the mechanism of millimolar toxicity, and to its correlation with *UnBiological*.

## Proposed mechanism of correlation of *Ub* with millimolar toxicity

### Selection against protein binding of metabolites

The observations above that many molecules interact with many cellular targets at millimolar concentration, and that these are likely to have significant biological effects, raises an obvious question. If many molecules can interact with many proteins at low millimolar levels, and such interaction has adverse effects on the cell, and many metabolites are present in the cell at low millimolar concentration, then why does the cell not poison itself with its own metabolites?

A plausible explanation is that the proteins (and other large molecular constituents of the cell) have evolved to avoid interference from the cell’s normal constituents. A protein that needs to interact with (say) an α-amino acid will evolve a binding site for that α-amino acid. A protein that does not require interaction with an α-amino acid for its function may nevertheless have a low affinity binding site for an α-amino acid in one of its conformers by chance. If this low affinity binding site has an adverse effect on the cell, then it will be selected against. In short, any non-specific interaction of the cell’s normal constituents will be selected against just as there will be positive selection for beneficial interactions.

Thus we would expect any binding site or pocket on a protein that could bind an amino acid to be selected against unless that interaction provided a beneficial effect on the function of the protein. Any compound that ‘looked like’ an amino acid (i.e. had similar chemical groups arranged similarly in space) would therefore also not find binding sites on that protein. Similarly there would be selection against random or fortuitous binding sites for the chemical features present in sugars, lipids, phosphate esters and other common structures in metabolism. However there would be no selection against low affinity, random binding to flurocarbons, organosilicon compounds or other chemicals quite different from anything normally in a cell. These, therefore, would be free to bind to any cellular protein if, by chance, a binding site happened to exist for them. The larger the segment of the xenobiotic that was unlike the chemistry of life, the greater the potential affinity for such a non-canonical binding site. The association of *UnBiological* with toxicity shown in Table 1 is therefore a consequence of the failure of biochemistry to be selected to avoid random binding of chemicals that the cell does not usually encounter.

### Testing the hypothesis with molecular docking

**Fig. 7:**
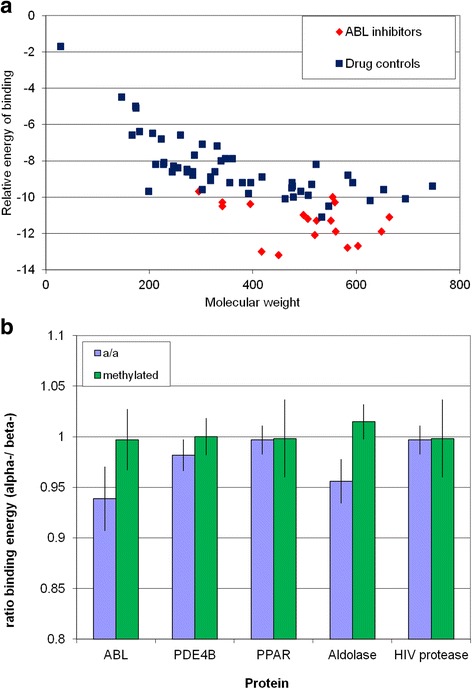
Docking small molecules with entire protein structures. **a**. Binding of 18 known ABL inhibitors, compared to the binding of 56 drugs or natural products not reported to have any effect on ABL kinase activity. Y axis: Vina output binding energy. X axis: molecular weight. **b**. Comparison of the predicted binding energy of 15 alpha amino acid and their alpha-N methyl alpha-carboxymethyl derivatives with the binding energy of equivalent beta amino acids and amino acid derivatives to ABL, Aldolase, HIV protease, PDE2b4b and PPAR gamma structures.. Excluded amino acids were: Glycine, which has no beta amino acid, beta alanine which is a metabolite in its own right and so was excluded, beta aspartate and asparagine which are the same as alpha aspartate and asparagines, and beta threonine which is likely to be unstable and so not a realistic chemical structure. Error bars are 95 % confidence limits (1.98*standard error of the mean)

Such a hypothesis has not been tested experimentally as far as I know, except in so far as low affinity binding of small molecules to proteins is commonly observed as noted above, although it has been observed that D-amino acids are mildly toxic to a wide range of microorganisms compared to their L-enantiomers [73]. In principle the low millimolar binding of small ligands to proteins could be tested computationally using molecular docking software, by trying to dock molecules known to *not* be ligands for a protein to that protein. Large-scale protein docking exercises do show that the majority of small molecules dock to target proteins with low millimolar or high micromolar affinity (for example [74–76]). Unfortunately, the low-affinity predictions of these exercises are unreliable (which is why they are usually ignored). As we do not know where the ‘binding site’ for a non-ligand might be, the test non-ligand must be docked to the whole protein, This provides such a large number of potential interactions that the software cannot reliably discriminate actual likely binding sites from implausible ones. Figure 7a illustrates this, docking 56 drugs with the ABL receptor. There is a strong trend for larger molecules to be predicted to have a higher affinity for ABL (which is plausible), but the largest molecules are predicted to bind as tightly as some *bona fide* inhibitors, despite having no known inhibitory effect on the enzyme.

If we confine ourselves to comparing molecules of the same size and atomic constitution, then some of the artefactual results shown in Fig. 7a might be avoided. Figure 7b shows the comparison of the predicted energies of binding of α-amino acids compared to β-amino acids to five mammalian proteins, selected to represent a mix of functional classes of proteins for which multiple structures and many authentic ligands were known. α-amino acids are core metabolites in mammals, β-amino acids are not part of normal mammalian metabolism with a couple of exceptions. For some but not all proteins tested, α-amino acids are predicted to bind with lower affinity than β-amino acids, as predicted by the hypothesis. The exceptions found here are HIV protease (which would be expected to bind amino acids, as they are related to its substrate) and PPAR-γ (for which I have no explanation). Repeating this exercise with more sophisticated models that took the dynamics of proteins as well as ligands into account (e.g. [77, 78]) might produce more useful results.

### Conflict with pharmaceutical experience

The suggestion that molecules that are *not* like biological molecules are more likely to be toxic appears paradoxical to the pharmaceutical chemist, as many drugs have potent (and hence potentially toxic) effects precisely because they are close molecular mimics of known metabolites. Thus, steroid drugs are potent precisely because they mimic natural steroids, dideoxyribonucleotides block viral DNA synthesis because of their mimicry of normal nucleosides [79, 80], penicillins mimic peptidoglycan components [81], and so on. However these molecules have been selected by evolution or by chemists to both mimic a specific biological effector *and* not to have any other effects than their target pharmacology. It is a truism of drug discovery that achieving this combination is extremely hard, and that unexpected or ‘off-target’ effects are a common cause of failure in drug discovery and development programmes [82–84]. Some of these effects are due to the close structural similarity between members of families of proteins, so that a drug selected to bind with high affinity to one target will be likely to bind to another, structurally similar target. However other ‘off-target’ effects are not obviously related to the known structural similarity of the ‘off-target’ proteins [85]. Yamanishi et al. [86] suggest that this is because small regions (equivalent to the Fragments used in my analysis) confer protein binding. A substantial fraction of the effort in drug discovery programmes is tailoring the specificity of the candidate drug to bind to a small number of targets, and many launched drugs actually bind to more than one protein family [87–90]. Drugs are therefore a special case, the result of extensive selection by man to fit with biology. The same explanation is true for the observation that chemicals that are not metabolites but fall within ‘Biochemical Space’ have a higher chance of being toxic even in the absence of selected pharmacology [4].

### Detoxification and resistance

A second apparent conflict with pharmaceutical experience is that organisms can and do tolerate a wide range of compounds that are toxic through tolerance, detoxification, and resistance mechanisms. The first two of these are less important to my general thesis than they might appear, and the third actually supports it.

Tolerance to a toxin or drug is almost invariably caused by changes in the organism’s physiology to compensate for the action of the drug or toxin. This is classically true of pharmacological agents such as alcohol, nicotine or heroin, but also to classic toxins such as arsenic. Mechanisms that oppose the effect of the drug or toxin are induced to restore a more normal physiological state. This is unrelated to the mechanism of intoxication in the first place.

Detoxification is a broad approach to removing toxins from an organism. It usually relies on enzymes (such as the CYPs in mammalian liver [91]) or transporters (such as the PGP family [92]) with very broad substrate specificities. It can also involve physical separation of the toxin into a defensive cell compartment. Compartmentalization is a common strategy for cells to sequester damaging metabolic chemistry from cell components that that chemistry might damage (e.g. oxidative phosphorylation in eukaryotes, anamox in prokaryotes). Sequestering misfolded proteins, damaged cell components or toxins can be seen as a form of ‘internal exile’, analogous to the export of these materials.

Acquisition of resistance be through one of two broad mechanisms. Detoxification mechanisms can be increased, often by mutation that increases expression of the relevant protein – this is a common mechanism of acquisition of drug resistance in cancer cells and in bacteria ([92, 93]. The other mechanism is for targets of the toxin to mutate so that they no longer bind the toxin. This is common for drug resistance [94–96]. It is not expected to apply to low potency, low molecular weight toxins, because (following my argument above) many, possibly most proteins would have to be mutated to evade toxicity. The mechanism of organisms’ resistance to chemicals *other* than drugs has not been reviewed systematically, so we do not know if this prediction is true.

## Conclusion

I have shown above that molecules that contain segments that are not similar to common components of metabolism are more likely to show toxicity at millimolar levels than compounds that have chemistry similar to life. I relate this to the widespread observation that many chemicals bind to many proteins at low millimolar levels, and that this can materially affect the function of those proteins.

This observation is an explanation for the observation that the chemistry of life occupies a small corner of the chemical space. In order to function, the components of the cell must interact with each other appropriately, both with functional interactions between the macro-molecules and metabolites of the cell and with the *absence* of unwanted interactions. Each new chemical added to metabolism requires adaptation of the whole proteome to accommodate the new chemical. Once a complex, self-perpetuating metabolism has evolved, adding to it will be an increasingly demanding evolutionary task, not an impossible one but one that the pragmatic mechanisms of evolution will tend to avoid.

This finding has two implications. Firstly, *UnBiological* could be used as a measure of the chance that a new molecule is toxic. Such broad toxicity predictions are less useful than predictions of specific mechanisms of toxicity, and *UnBiological* specifically does not provide a mechanistic explanation. It is also only as statistical estimate. From the data analysed here, *Ub*_*5*_ or *Ub*_*6*_ could be used to give an order-of-magnitude estimate of the potency of a low-potency toxin, but would say nothing about high potency toxicity. It is possible that coupling *Ub* with other measures [4] might give more accurate estimates. As an initial screen for ‘drug-like’ properties [97–100], however, such a statistical indicator could find a use.

In this application of predicting toxicity, a strong limitation of the analysis presented here is that it takes no account of the concentration of metabolites in the cell. Metabolic intermediates present at nanomolar concentration are given the same weight in the *Ub* calculations above as common components such as glucose or glycine. One would however expect the selective pressure on proteins to avoid binding glucose to be much stronger than the pressure to avoid binding metabolites present at nanomolar concentrations.

An extension of this work would therefore include a concentration term in the calculation of *UnBiological*. This would include two components – consideration of the differing metabolomes of different cells or organisms, and quantitative consideration of the concentration of metabolites in an organism. In this study, a single collection of metabolites (“core metabolism”) was used to define UnBiological. I expect that predictions of toxicity based on the actual intracellular metabolome of a specific species would be more accurate for that species (and less accurate for other species) than this generic approach. This is however a substantial undertaking, involving re-calculation of most of the comparisons presented here for each species, and so has not been attempted in this paper: my goal here is to show that this approach is theoretically and practically interesting. It might also be valuable to weight the contributions of metabolies to the Ub calculation according to their intracellular concentration, although this is fraught with difficulty as intracellular concentrations of metabolites are very hard to measure, and in any case are modulated by the protein binding that this study postulates occurs promiscuously and universally. Future work could also explore the size of the overlap necessary to define *Ub*: again, this would be doable, but time-consuming, and so has been left for future work.

The second implication of this work is in the field of metabolic engineering and synthetic biology. Engineering an organism to produce a new chemical or execute a new metabolic pathway has been thought to require the expression of suitable enzymes to make the chemical and any intermediate or precursor molecules at sufficient concentration, efficiency, and from suitable feedstock. The rest of the cellular machinery is generally viewed as a ‘chassis’ on which to attach these changes [101–103]. For chemicals or gene products produced at low concentrations this is likely to be true [104]. However if the goal of the engineering is to produce a chemical at substantial levels [105], then the analysis in this paper suggests that many aspects of the cell must be engineered, especially if the chemical to be produced is very different from one usually present in the cell.

## Methods

### Toxicity data

Databases of molecular structures and biological endpoints were collected from literature sources, as listed in Table 3. Data was filtered to collect toxicity endpoints that were, as far as practical, the same endpoint for different studies on the same organism. Data sets were collected that

- provided a quantitative half-effect concentration estimates (i.e. not single concentration toxicities)
- provided data on at least 50 compounds from diverse chemical families
- were available from a small number of sources (for practical reasons, data sets of 200 compounds studied in 200 papers were not used)
- were from species with recognised use in toxicity testing.

All EC_50_ values were as reported in the relevant papers or databases except those for *Saccharomyces cereviseae*, where EC_50_ values were calculated from the raw inhibition data downloaded from [106]. The *Saccharomyces* data set was filtered to exclude organometallic compounds, to exclude mixtures or salts other than halogen or alkali metal salts, compounds for which growth inhibition at the highest concentration was <50 % or for which the growth inhibition at the lowest concentration was >50 %, and compounds for which the range of calculated EC_50_s across the 13 strains tested in this data set (calculated as [maximum(EC_50_)-minimum(EC_50_)]/average (EC_50_)) was >1. The resulting data set represented well defined organic compounds with EC_50_s within the experimentally measured concentrations and consistent toxicity across a range of *Saccharomyces cereviseae* strains.

Chemical structures were collected as. MOL files, and compiled into an SDF file for processing.

### Molecules of metabolism

The chemical space of metabolism was taken as all the molecules shown on the printed version of Part A (intermediary metabolism) of the Roche/Expasy metabolic chart, with the exception of the steroid hormones. Steroid hormones were omitted because they represent many elaborations on the same core (sterol) structure, and do not add significantly to the diversity of chemical types. The chemical space of 611 metabolites is referred to as ‘core metabolism’ in this paper.

### Generation of ‘all’ molecules in chemical space

The space of all possible chemical structures was explored by the program Combimol [5]. In brief, the program generates chemical structures based on SMILES strings [107]. The program aims to generate all the molecules of a specified size (defined as number of non-hydrogen atoms), using a subset of C, N, O, S, P or Si that is specified by the user. ‘All molecules’ are here defined as all 2-D chemical structures that have a bonding pattern consistent with the valencies of the atoms used: four bonds for C and Si, 3 for N, 2 for O, 3 or 5 for P, 2, 4 or 6 for S. For the work described in this paper, silicon was excluded from this list, and only P(V) and S(II) and S(VI) (sulphate) were used, consistent with those elements’ use in metabolism.

The SMILES language is a simple, text-based method for coding chemical structures [107]. The program starts by generating an exhaustive list of all possible linear atom strings up to the desired size: if the maximum size was 4, it would generate CC, CCC, CCCC, CCCN, CCCO, CCCP, CCNC etc.. It then replaces single bonds with double bonds in any position allowed by the elements (CCC = C, CC = CC etc.). It then generates cyclised versions of these strings (C1CCC1, where the two ‘1’ symbols represent atoms that are connected – the reader is directed to [107] for a further description of the SMILES language). A molecule can have up to two ring systems, including fused rings. These form unbranched ‘core’ molecules. In a final step, the ‘core’ molecules are joined to each other to form branched molecules: thus CCC and CC could be joined to form CC(C)C.

A number of rules are included in this process to remove atom combinations that would be unstable (such as COOOC).

As described in [5] the program is not completely exhaustive (it under-represents quaternary carbon centres, for example): however I estimate that it generates over 90 % of the molecular structures that are consistent with the rules set provided. The program, and a new, more systematic version, which was not used in this work but will be for future work, is available for non-commercial applications from the author.

### Molecular matching and similarity

I define here the terms for molecular structures and matching used in this paper. These are not significantly different from how these terms are used in the general chemical literature, but are laid out explicitly here to avoid confusion.

- A molecule is a set of atoms connected by bonds, in which all the atoms’ valencies are filled. It is assumed that all valencies that are not explicitly linked to another atom in the description of the molecule are filled with hydrogen atoms. (“molecule” here is completely consistent with the common understanding of the term, and so will not be capitalised: I define it here solely for completeness).
- A Fragment is a set of atoms connected by bonds, in which the valencies of at least one of the atoms are not filled. A Fragment therefore represents part of the structure of a molecule, and not a real physical entity.
- Substructure. Molecule or Fragment A was said to be a substructure of molecule or Fragment B when all of the non-hydrogen atoms and all the bonds of molecule or Fragment A could be overlaid on molecule B in at least one position.
- Overlap. Molecule or Fragment A and molecule or Fragment B are said to have an N-atom overlap when the largest Fragment which is a substructure of molecule A *and* of molecule B has N atoms.

To identify matches and Overlaps between molecules and Fragments, I used a 2D fragment-based molecular descriptor system that has previously been described [7, 8], and proven effective in building models to predict toxicity outcomes. In summary, I generate an exhaustive set of Fragments from all the molecules used in this study as follows. For each pair of molecules, the maximum common structure (Maximum Common Subgraph – MCS) is found by ‘overlapping’ the 2D molecular structure. The MCS between each of these Fragments and between each Fragment and each original molecule is then computed to generate further Fragments. This is repeated until no new Fragments are found. The result is a list of all the molecular Fragments that are present in two or more of the molecules in the set. Fragments of 1 or 2 atoms are ignored.

Molecular descriptors of a molecule are then computed by matching a set of these molecular Fragments to that molecule, and counting the number of distinct ways that a Fragment can be exactly mapped onto a molecule. A descriptor is an integral count of the number of occurrences of a Fragment in a molecule. The molecule as a whole is described by the pattern of Fragment descriptors.

Molecular matching and molecular Fragment generation were performed by software build by Amedis Pharmaceuticals Ltd. (see [7, 8] for details) and kindly provided by Dr. Antranig Basman. Conversion of data files for transfer between programs was done with a number of small programs written specifically for this project in Qbasic, and compiled with the QB64 compiler [108]. All programs other than MolDescrip are available from the author on request, and source code for programs other than those originating from Amedis Pharmaceuticals are also available. General chemical database manipulation was done using the CambridgeSoft ChemBioOffice suite version 12.0, under site licence to MIT. All work was done on standard PCs running various versions of Windows depending on their age.

### Docking

Docking was done using AutoDock Vina [109]. The ‘binding site’ was defined as the entire surface of the protein for all the proteins. Potential ligands were docked to a number of structures for each protein, as listed in Table 4. Ligands were docked using default parameters except for ‘Exhaustiveness’, which was set = 100. The binding energy of a ligand to a protein was taken as the maximum (most negative) binding energy of any ligand conformation to any site on any of the tested protein structures.

### Calculation of ‘Unbiological (Ub)

‘Unbiological’ is a measure of the size of a region of a molecule that is not represented in metabolism. In this paper ‘metabolism’ is taken to be the set of ‘core metabolism’ molecules defined by the 611 chemicals listed in the Roche/Expasy metabolic map, as described above (section Molecules of metabolism).

What is meant by ‘not represented’ depends on the size of the Fragment that is being considered. If we only require one atom similarity between a metabolite and a test molecule, then clearly almost all molecules can ‘match’ a core metabolite. Thus *Ub* depends on our definition of similarity.

In this study, I define *Ub* as follows: Fig. 3 illustrates this process. The metabolites of ‘core metabolism’ are broken into N-atom fragments. A region is ‘unbiological’ if it does not contain an exact match to any of the fragments generated from core metabolism. Thus in Fig. 3, three metabolites (top left) and three test chemicals (top right) generate 12 Fragments, of which 9 (bottom left) completely match the original metabolites. The three Fragments not found in metabolites (bottom right) are *UnBiological*. These are matched to the target molecules (bottom right). The size of the largest *UnBiological* fragment that can be matched to a test molecule is its *Ub* value.

This is a measure of the size of a sub-region of a molecule that has an arrangement of atoms unlike an arrangement found in biology.

As noted in the text, this depends on the original set of biochemicals used as a definition of ‘biochemistry’. The use of central metabolic pathways is convenient, but could be improved.

## Ethics and consent

This work involved no human or animal experimentation, and so no ethical or other consent was relevant.

## Availability of data and materials

The complete data set of Ub values, toxicity endpoints for the chemicals analysed in this study is available for download from LabArchives (https://mynotebook.labarchives.com/), at DOI 10.6070/H4VQ30PJ (direct URL for download of the spreadsheet https://mynotebook.labarchives.com/share_attachment/Bains_Data/MTkuNXwxNTAzOTUvMTUtMy9UcmVlTm9kZS83NjQwNTkxNDl8NDkuNQ==). A ZIP file of the MOL files for rhe chemicals used in this study can be downloaded from https://mynotebook.labarchives.com/share_attachment/Bains_Data/MTkuNXwxNTAzOTUvMTUtNC9UcmVlTm9kZS8yMzAwNTAxMjU0fDQ5LjU=. The programs used for this specific analysis were proprietary to Amedis Pharmaceuticals Ltd (Cambridge, UK), when that company existed, and are available as compiled code only: consequently they are not available for general use. However similar chemical matching functionality can be found in RDKit, (http://www.rdkit.org/docs/index.html), which is an open-source, Python based platform.

## Figures and Tables

**Fig. 8:**
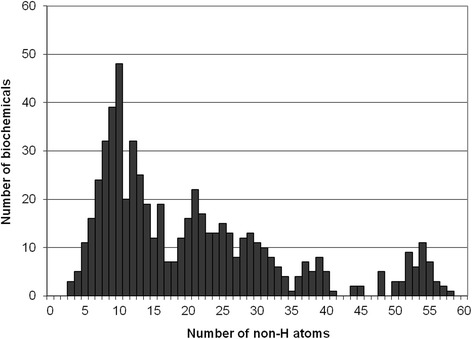
Size distribution of the molecules in the ExPasy/Roche biochemicals pathway map of central metabolism [10]

## Appendices

### Appendix 1: Method for calculating the likelihood that a random sampling of a chemical space will contain a specified substructure of specified size

#### Random sampling of chemical space

The likelihood that a set of molecules *M* contains at least one target molecule *T* containing the substructure *S* is not simple to estimate, for four reasons.

Firstly, some substructures are much more abundant in a ‘random’ chemical space than others. The structure C-C can be linked to 6 other atoms, in molecules ranging from C_2_H_6_ up in size. The structure C≡N can only be linked to one other atom, even though it has the same number of non-hydrogen atoms. Thus CC is going to lead to more structural possibilities than C≡N, and hence occur more frequently. The relative abundances of different substructures are hard to predict *ab initio*.

Secondly, substructures are not independent, and so two examples of *S* in *M* cannot be treated as independent events. For example, if we find the structure C=C-N-C in *M*, our expectation that we also find C-N-C-C is higher than if we do not find C=C-N-C, because the two substructures overlap.

The probabilities of finding a specific substructure is also dependent on the atoms and bond types used to construct the chemical space to be explored. Finding N-N is more likely in a chemical space that contains hydrazines.

Lastly, the maximum number of fragments *S* that could match *T* is A_T_-A_S_, where A_T_ is the size of *T* and A_S_ is the size of *S*. However it might be less than this, due to symmetry and duplication within *T*. For example, cyclooctane only matches the three-atom fragment C-C-C, and no other three-atom fragment.

These multiple, interrelated contingencies lead me to estimate the chances of finding *S* in *M* empirically by simulation. I generated a large set of molecules generated using Combimol under the same rules as described in the main text. From this I selected a random subset *M* (random subsets were selected using the Excel RAND () function). Sets *M* of 50, 200, 611 and 2000 molecules of 7, 14 and 21 non-H atoms were probed with the fragment sets of 3, 4, 5 and 6 atoms, using the same approach as in Fig. 2 of the main paper. Multiple runs of the Fragment set against different, randomly selected *M* were run, and average and standard deviation of the number of *S* not found were recorded. The results are shown in Table 5.

We can seek an empirical model for the fraction of the set of all fragments of size A_F_ that are not found in a set of molecules *M* of whose geometric mean size (measured in number of non-hydrogen atoms) is A_M_ and containing N_M_ molecules. I define this fraction as Fragments Not Found (FNF). Inspired by the similarity of the curves of FNF vs N_M_ to the curves of enzymes reaction rates following Michaelis Menten kinetics, I find that the numbers in Table 5 are roughly modelled by

1 $$ FNF=1-\frac{N_M}{e^{\left(0.88*{A}_F+\frac{0.35}{A_M}*{A}_F^2\right)}+{N}_M} $$

Where A_F_ = number of atoms in the fragments, N_M_ = number of molecules in *M*, A_M_=geometric mean of the size of molecules in *M*. The exponential in A_F_ and A_F_^2^ is expected from the equations for the numbers of molecules in an organic chemical space as a function of molecular size [5].

We can use Eq. 1 to predict how many fragments would not match a set of chemicals with the same size distribution as the molecules in the central, primary metabolism of life, described in [110] and shown in Fig. 8. These are the “Control” frequencies of un-detected fragments shown in Fig. 2

**Table 5:** Modelled expectations of fragment matching to sets of chemicals

		A_M_	7 atoms				14 atoms				21 atoms			
A_F_	N_F_	N_M_	50	200	611	2000	50	200	611	2000	50	200	611	2000
3	62		0.356	0.177	0.076	0.024	0.292	0.136	0.041	0.019	0.245	0.084	0.048	0.012
4	318		0.571	0.273	0.101	0.021	0.465	0.202	0.063	0.022	0.356	0.127	0.039	0.011
5	1363		0.832	0.561	0.321	0.119	0.702	0.448	0.240	0.092	0.611	0.335	0.164	0.064
6	9240		0.975	0.904	0.771	0.529	0.899	0.760	0.581	n/m	0.852	0.674	0.476	n/m

A_F_ – number of (non-hydrogen) atoms in the fragment set. N_F_ – number of fragments in the chemical space containing A_F_ atoms. N_M_ – number of test molecules in the test set. A_M_ – number of atoms in the molecules in the test set. Cells show the fraction of Fragments that are *not* found in at least one of the Molecules in the test set. n/m = not modelled

.

### Appendix 2: Detailed description of the method and software used to calculate the ‘UnBiological’ parameter

#### Calculation of UnBiological (*Ub*)

The following is a description of how the UnBiological (*Ub*) measure was generated. Note that this is not the most efficient way of generating this parameter that could be defined, but it uses the software available. I strongly suggest that any researchers who wish to replicate and extend this work develop their own software using an open-source code base like RDKit, whose internal functioning is accessible.

Software used is:

- FRAGGEN. Inputs a set of. MOL files and outputs a set of MOL files containing all the Fragments that can be generated from the input files that match at least two of the input molecules. A ‘Fragment’ in this regard is a connected group of atoms that is common to at least two of the molecules in the input data set.
- MOLDESCRIP. Inputs a parameter file, a set of Fragment MOL files as *descriptors*, and a set of target MOL files as *targets*. Identifies all the matches where a Fragment overlaps a target MOL file.
- Conversion files, to convert input and output files from FRAGGEN and MOLDESCRIP into forms usable for this project.

Steps performed.

1. Generate all the Fragments of metabolites by running FRAGGEN on the set of molecules in the ExPasy database. This is the set of Fragments MetabFrags.
2. Generate all the Fragments of the molecules to be tested, by running FRAGGEN on those molecules. This is the set of Fragments AllFrags. (The set of ‘all molecules’ should include the metabolites)
3. Sort the MetabFrags by size.
4. Repeat the following steps for each class of MetabFrags having MM atoms:

4.1 Find the AllFrags fragments that have no MetabFrag Fragments that overlap them, by running MOLDESCRIP with MetabFrag descriptors and AllFrag targets. This generates a list of Fragments from AllFrags that have no overlap with a metabolite of MM atoms
4.2 Sort these by size. This gives sets of AllFrag Fragments that a) have no overlap with metabolites of size MM and b) are size AA.
4.3 For each of these:
  4.3.1 Find whether each fragment is present in the target set of molecules, by running MOLDESCRIP with the selected set of AllFrag fragments as *descriptors* and the set of target molecules as *targets*.
  4.3.2 Any molecule with a match to a fragment that a) has no overlap with metabolites of size MM and b) is size AA has a *Ub* score of ≥ AA.
  4.4 Repeat for increasing values of AA until 95 % of the target molecules are covered.

In this paper, all transfers between programs and data reformatting was performed manually. An alternative approach would be to directly search for overlap of each of AllFrags with all of the Metabolites to find AllFrags that are *UnBiological*. This is impractical with the software to hand, as there are >30,000 AllFrags, and MOLDESCRIP generates inconsistent output file formats with more than ~4000 *descriptors*.

### Appendix 3: Statistics on the distribution of UB5 and Ub6 and toxicity endpoints for all the data sets analysed in this study, together with a short commentary

#### Distributions of Ub and toxicity measures

**Table 6:** Summary statistics on the toxicity and UnBiological values for different biological endpoints

Endpoint	Number	Correlation with Toxicity		Endpoint distribution (log molar)						Ub5 distribution						Ub6 distribution					
		Ub_5_	Ub_6_	mean	median	Std. dev	skew	max	min	mean	median	St. Dev	skew	max	min	mean	median	St. Dev.	skew	max	min
Trout 24 h	186	-0.230 **	-0.337 ***	-5.73	-5.64	1.39	-0.14	-0.99	-10.04	7.58	7	1.92	0.01	**11**	**2**	8.94	9	1.91	-0.74	**12**	**2**
Trout 96 h	181	-0.419 ***	-0.516 ***	-5.73	-5.78	1.77	0.76	-0.23	**-10.88**	7.58	7	**1.94**	0.01	**11**	**2**	8.94	9	1.92	-0.76	**12**	**2**
Pteronarcys (24 h)	52	-0.433 **	-0.385 **	-6.25	-6.83	1.27	0.75	-3.24	-7.98	**8.52**	**8**	1.70	0.15	**11**	6	9.71	**10**	1.60	-0.37	**12**	6
Pteronarcys (96 h)	52	-0.456 ***	-0.369 **	-7.08	-7.58	1.44	0.61	-3.71	-9.55	8.44	**8**	1.67	0.22	**11**	6	9.65	**10**	1.58	-0.29	**12**	6
Bluegill (24 h)	157	-0.149	-0.215 **	-5.71	-5.63	1.41	-0.21	-2.31	-9.61	7.92	**8**	1.82	0.22	**11**	4	9.30	9	1.65	-0.28	**12**	4
Bluegill (96 h)	172	-0.216 **	-0.276 ***	-5.88	-5.84	1.48	-0.16	-2.31	-10.20	7.85	**8**	1.82	0.23	**11**	4	9.23	9	1.70	-0.33	**12**	4
Gammarus (24 h)	113	-0.437 ***	-0.208 *	-6.17	-6.35	1.46	-0.16	-3.52	-10.73	8.19	**8**	1.71	0.43	**11**	6	9.64	**10**	1.46	-0.16	**12**	6
Gammarus (96 h)	132	-0.407 ***	-0.205 *	-6.56	-6.66	1.63	-0.12	-3.20	-10.82	8.14	**8**	1.71	0.46	**11**	6	9.57	9	1.47	-0.12	**12**	6
Fathead minnow	578	-0.311 ***	-0.308 ***	-3.83	-3.82	1.38	-0.15	-0.04	-9.38	5.59	5	1.41	**1.07**	**11**	**2**	7.07	7	1.75	0.19	**12**	**2**
Rat oral	814	-0.441 ***	-0.372 ***	-2.40	-2.27	0.91	-0.99	-0.43	-6.98	6.57	6	1.64	0.40	**11**	**2**	8.11	9	2.14	-0.34	**12**	**2**
Mouse oral	398	-0.199 ***	-0.191 ***	-2.56	-2.47	0.82	**-1.30**	-0.65	-6.34	6.96	7	1.43	-0.02	**11**	**2**	8.80	9	1.86	-0.57	**12**	**2**
Rat IP	170	-0.214 **	-0.147	-3.03	-2.88	0.86	-0.66	-0.90	-5.61	6.85	7	1.35	0.24	**11**	**3**	8.64	9	1.71	-0.31	**12**	**3**
Mouse IP	290	-0.180 **	-0.161 **	-3.21	-3.02	1.04	-0.93	-0.99	-7.50	6.85	7	1.45	-0.09	**11**	**2**	8.50	9	1.98	-0.46	**12**	**2**
AMES	163	-0.316 ***	-0.518 ***	-4.72	-4.25	1.67	-0.51	-1.52	-9.88	6.33	7	1.33	0.07	**11**	**2**	8.40	9	2.09	-0.49	**12**	**2**
CPDBAS rat	519	-0.198 ***	-0.191 ***	-4.18	-4.19	1.42	-0.24	-0.47	-9.85	6.36	6	1.35	-0.09	**11**	**2**	8.03	8	2.08	-0.23	**12**	**2**
CPDBAS mouse	402	-0.145 **	-0.198 ***	-3.62	-3.54	1.18	-0.60	-0.53	-9.32	6.19	6	1.43	-0.18	10	**2**	7.85	8	2.21	-0.32	**12**	**2**
CPDBAS hamster	44	-0.430 **	-0.351 *	-4.46	-4.53	0.99	0.43	-1.89	-6.05	5.95	6	1.51	**-0.64**	9	**2**	7.32	8	**2.43**	-0.17	**12**	**2**
Drosophila	139	-0.397 ***	-0.337 ***	**-1.43**	**-1.29**	0.94	-0.82	0.23	**-4.80**	5.82	6	1.35	0.17	10	**2**	7.32	7	2.07	0.00	**12**	**2**
Lemna - non-Herbicides	149	-0.428 ***	-0.502 ***	-4.85	-5.12	1.47	0.85	-0.24	-8.20	7.01	7	1.50	-0.37	**11**	**2**	8.68	9	1.94	-0.87	**12**	**2**
Lemna - Herbicides	174	-0.392 ***	-0.428 ***	-6.21	-6.08	1.65	-0.19	-2.99	-10.23	8.02	**8**	1.63	0.27	**11**	4	9.56	9	1.77	-0.02	**12**	4
Tetrahymena	334	-0.408 ***	-0.448 ***	-3.51	-3.59	0.97	0.10	-1.07	-5.82	5.62	5.5	1.03	-0.01	**8**	**2**	7.16	7	1.55	-0.11	**12**	**2**
Chlorella	91	-0.578 ***	-0.738 ***	-2.86	-3.16	1.47	0.43	**1.06**	-6.10	6.15	6	1.74	0.86	**11**	**2**	7.62	7	1.77	-0.43	**11**	**2**
Scenedesmus	63	-0.237	-0.467 ***	-5.80	-5.90	1.61	**1.24**	0.10	-8.04	6.27	6	1.15	0.10	9	3	8.17	9	1.53	**-0.92**	**11**	3
Yeast	253	0.095	-0.014	-4.69	-4.56	**0.44**	-0.61	-4.01	-5.77	6.76	7	1.02	0.01	10	4	8.96	9	1.42	-0.01	**12**	5
NCI	768	-0.113 **	-0.137 ***	-5.03	-4.67	1.38	-1.19	-2.03	-10.02	7.04	7	1.07	0.81	**11**	4	9.14	9	1.33	-0.20	**12**	5
HERG	229	-0.062	0.179 **	-5.50	-5.42	1.27	-0.09	-2.36	-8.59	7.14	7	1.01	-0.14	10	4	9.67	9	1.29	-0.03	**12**	5
Oestrogenic	131	-0.024	-0.342 ***	-5.62	-5.32	**1.78**	-0.47	-2.52	-9.65	6.16	6	0.95	0.49	10	4	8.97	9	1.24	-0.06	**12**	6
Tadpole narcosis	141	-0.043	-0.267 **	-2.47	-2.37	1.15	-0.32	-0.19	-5.33	**5.01**	**5**	1.32	0.82	10	**2**	**6.16**	**6**	1.82	**0.48**	**11**	**2**
COX-2	107	-0.069	-0.149	-6.36	-6.52	1.51	0.48	-3.00	-8.70	7.72	**8**	**0.56**	0.36	10	**7**	9.20	9	**0.68**	-0.08	**11**	**8**
Antihistamine	61	-0.097	-0.0126	**-7.99**	**-8.13**	1.27	0.84	**-4.05**	-10.59	6.92	7	0.76	0.61	9	6	**10.2**	**10**	1.26	-0.23	**12**	7

Left column, biological endpoints correlated in this study. Column 2 – number of molecules in the data set for that endpoint. Columns 3 and 4, correlation of the endpoint with UnBiological measures Ub5 and Ub6, as per Table 3. The rest of the table lists summary statistics on the toxicity endpoints (all in Moles/lirte for solution studies, moles/kg for animal studies), Ub5 or Ub6. Listed are mean, median, Standard deviation (St. Dev.), Skew (a measure of the asymmetry of the data - positive skewness indicates a distribution with an asymmetric tail extending toward more positive values, negative skewness indicates a distribution with an asymmetric tail extending toward more negative values), and maximum (max) and minimum (min) values

The maximum and minimum values in each column are underlined for ease of comparison. There are more minimum values for the biological endpoint in the pharmacologically defined endpoints Cox-2 and antihistamine, as would be expected as these molecules have been selected specifically for pharmacological potency (i.e. for low EC_50_ values). The molecular set used for Tadpole Narcosis has more minimum values for Ub_5_ and Ub_6_ statistics than might be expected by chance, suggesting that the set of molecules tested for narcosis induction in tadpoles is biased with respect to the other sets used in this study. Other sets are generally not obviously different from each other, and specifically the Ub_5_ and Ub_6_ statistics for HERG, Oestrogenic potential. COX-2 inhibition and Antihistamine efficacy appear be broadly similar to the Ub_5_ and Ub_6_ statistics for the molecule sets analysed for toxicity endpoints

## Footnotes

1 No hits for searches for this structure in the Dictionary of Natural products.

2 There were not enough compounds in the wider set to make any statistical conclusion on the correlation, so it is not included in Table 2.
